# Infective Endocarditis in the U.S., 1998–2009: A Nationwide Study

**DOI:** 10.1371/journal.pone.0060033

**Published:** 2013-03-20

**Authors:** David H. Bor, Steffie Woolhandler, Rachel Nardin, John Brusch, David U. Himmelstein

**Affiliations:** 1 Department of Medicine and Division of Infectious Diseases, Cambridge Health Alliance/Harvard Medical School, Cambridge, Massachusetts, United States of America; 2 School of Public Health, City University of New York, New York, New York, United States of America; and Department of Medicine, Cambridge Health Alliance/Harvard Medical School, Cambridge, Massachusetts, United States of America; 3 Department of Medicine and Division of Neurology, Cambridge Health Alliance/Harvard Medical School, Cambridge, Massachusetts, United States of America; Oxford University, Vietnam

## Abstract

**Background:**

Previous studies based on local case series estimated the annual incidence of endocarditis in the U.S. at about 4 per 100,000 population. Small-scale studies elsewhere have reported similar incidence rates. However, no nationally-representative population-based studies have verified these estimates.

**Methods and Findings:**

Using the 1998–2009 Nationwide Inpatient Sample, which provides diagnoses from about 8 million U.S. hospitalizations annually, we examined endocarditis hospitalizations, bacteriology, co-morbidities, outcomes and costs. Hospital admissions for endocarditis rose from 25,511 in 1998 to 38, 976 in 2009 (12.7 per 100,000 population in 2009). The age-adjusted endocarditis admission rate increased 2.4% annually. The proportion of patients with intra-cardiac devices rose from 13.3% to 18.9%, while the share with drug use and/or HIV fell. Mortality remained stable at about 14.5%, as did cardiac valve replacement (9.6%). Other serious complications increased; 13.3% of patients in 2009 suffered a stroke or CNS infection, and 5.5% suffered myocardial infarction. Amongst cases with identified pathogens, Staphylococcus aureus was the most common, increasing from 37.6% in 1998 to 49.3% in 2009, 53.3% of which were MRSA. Streptococci were mentioned in 24.7% of cases, gram-negatives in 5.6% and Candida species in 1.0%. We detected no inflection in hospitalization rates after changes in prophylaxis recommendations in 2007. Mean age rose from 58.6 to 60.8 years; elderly patients suffered higher rates of myocardial infarction and death, but slightly lower rates of Staphylococcus aureus infections and neurologic complications. Our study relied on clinically diagnosed cases of endocarditis that may not meet strict criteria. Moreover, since some patients are discharged and readmitted during a single episode of endocarditis, our hospitalization figures probably slightly overstate the true incidence of this illness.

**Conclusions:**

Endocarditis is more common in the U.S. than previously believed, and is steadily increasing. Preventive efforts should focus on device-associated and health-care-associated infections.

## Introduction

William Osler’s 1885 study of malignant endocarditis patients at Montreal General Hospital set a standard for clinical-pathophysiological correlation [1]. Subsequent studies using modern microbiological techniques and echocardiography have documented dramatic shifts in the profile of this disease [2]–[7] from predominantly sub-acute streptococcal infections of younger women with rheumatic heart disease to acute staphylococcal illnesses of older men with degenerative heart diseases or intravascular devices. These data have informed recommendations regarding prophylaxis and therapy.

Endocarditis incidence estimates have come from studies at referral hospitals [5] or from a single county or metropolitan area [8]. Since virtually all patients diagnosed with endocarditis are hospitalized, we analyzed a nationally representative sample of US hospital discharges between 1998 and 2009 to document the further evolution of this disease in the 21^st^ century.

## Methods

### Ethics Statement

Because the study involved only secondary analysis of data available in the public domain, it was exempt from ethics review.

We analyzed the Agency for Healthcare Research and Quality’s Nationwide Inpatient Sample (NIS) for 1998–2009 which provides hospital discharge data for about 8 million inpatient stays annually from a 20% sample of non-federal acute care hospitals [9] (http://www.hcup-us.ahrq.gov/nisoverview.jsp). The NIS provides weights to allow extrapolation to all US hospitalizations. Prior to 2009, the NIS included up to 15 diagnosis codes and 15 procedure codes from each patient’s hospital discharge abstract, using the International Classification of Diseases, 9^th^ Revision, Clinical Modification (ICD-9-CM). The 2009 NIS includes ten additional diagnoses for a total of 25.

The NIS also provides data on age, sex, race, expected source of payment, the income quartile of patients’ zip code of residence, disposition (e.g. home, another acute care hospital, sub-acute facility), hospital charges and hospital characteristics.

We considered a patient to have infective endocarditis (IE) if their record included any of the following diagnostic codes: 4210 (acute and subacute bacterial endocarditis), 4211 (acute and subacute infective endocarditis in diseases classified elsewhere – e.g. Q fever), 4219 (acute endocarditis, unspecified), 03642 (meningococcal endocarditis), 09884 (gonococcal endocarditis), 11281(candidal endocarditis) or 1154 (histoplasmosis endocarditis). We excluded endocarditis due to syphilis, rheumatic heart disease (without infection), lupus or other non-infectious causes.

The Appendix lists the ICD-9-CM codes we used to identify causative organisms, comorbidities, predisposing factors, procedures during hospitalization, and complications.

The ICD-9-CM does not include a specific code identifying culture-negative endocarditis. Hence, the lack of a bacteriologic diagnosis may sometimes indicate failure to code a known causative organism. To explore the characteristics of patients lacking a specific bacteriologic diagnosis we compared the characteristics of this group to patients for whom a causative organism was coded.

### Data Analysis

We first calculated the number of hospital stays for which a diagnosis of endocarditis was coded. Because about 9.9% of endocarditis hospitalizations ended with a transfer to another acute care hospital, we estimated the number of unique endocarditis cases by assuming that all transferred patients were coded as “endocarditis” at the receiving hospital, and subtracting them from our estimates. Such transferred patients were also excluded from calculation of death rates. (We could not identify other patients who might be duplicates in our data, i.e. those who were discharged and subsequently readmitted during a single episode of IE. Hence, our estimation of the number of IE cases may be slightly high). Additionally, we excluded the 0.2% of 2009 records in which endocarditis was coded but not among the first 15 diagnoses. We compared changes in endocarditis hospitalization rates between 1998 and 2009 using Census Bureau figures and the direct method to adjust for population growth and aging [10], [11].

We calculated the proportion of IE hospitalizations due to each organism in two ways: first, as a proportion of all IE hospitalizations and then as a proportion of these with at least one organism coded. We analyzed IE hospitalizations by gender, race and age group, as well as by hospital characteristics, length of stay (LOS), expected source of payment, hospital charges, and disposition. In addition, we examined pre-disposing factors, co-morbidities (see Appendix), and complications including valve replacement, death, myocardial infarction (MI), neurological complications or acute renal failure (ARF).

### Statistical Analysis and Considerations

We performed chi square tests to evaluate differences in proportions, and Cochran-Armitage tests to evaluate time trends. For continuous variables we used t tests to evaluate differences, and linear regression to evaluate time trends. Because the NIS sample size is large, most observed differences (and all of those we cite) are statistically significant (p<.0001) unless otherwise noted.

## Results

Hospital stays for IE rose from 28,195 in 1998 to 43,419 in 2009 (Table 1); this 54.0% increase exceeded the 13.1% growth in total hospital admissions (p<.0001). After adjustment for the 9.9% of IE hospital stays that ended in a transfer to another hospital within the NIS sampling frame, the number of unique endocarditis hospitalizations was 25,511 in 1998 (9.3 per 100,000 population) rising to 38, 976 in 2009 (12.7 per 100,000 population) (p<.0001). After adjustment for population aging and growth, endocarditis hospitalizations increased by 2.4% annually. Hence, the increase was not simply due to population aging.

**Table 1:** Number of Hospital Stays, and Hospitalized Cases with Diagnosed Infective Endocarditis, Mean Length of Stay and Hospital Charges, 1998–2009.

Year	Number of Hospital Stays	Number of Hospitalized Cases, Adjusted for Transfers	Mean Length of Stay(Days)	Mean Hospital Charges ($s)
**1998**	28,195	25,511	15.3	$45,542
**1999**	29,420	26,583	15.5	$50,752
**2000**	29,868	27,150	15.1	$57,693
**2001**	31,526	28,506	15.9	$64,445
**2002**	32,229	29,399	15.2	$69,853
**2003**	35,190	31,893	15.6	$85,395
**2004**	36,660	32,959	15.4	$81,759
**2005**	37,508	33.615	15.6	$86,515
**2006**	40,573	36,642	15.3	$90,529
**2007**	38,207	34,028	15.0	$99,559
**2008**	41,143	36,892	15.5	$114,576
**2009**	43,419	38,976	15.0	$122,204
**12 Year Total**	423,938	382,153	15.3	

Mean LOS was 15.3 days and varied little over the 12 years of the study (p = .55). Mean hospital charges more than doubled in real terms from $45,542 ($59,941 in 2009 dollars) to $122,204. Adjustment for transfers to other acute care hospitals would increase these figures by about 10%. Both LOS and charges were about four times higher than the average for all diagnoses, a ratio that did not change over time.

Hospital charges and LOS varied little by age among adults aged 18–69, but were lowest for those 70 and older and highest for children.

### Epidemiology

Table 2 displays demographic features of patients hospitalized with endocarditis. A majority (57.7%) were male. More than a third (36.4%) were age 70 and above. Most patients resided in zip codes with below average incomes. Most hospitalizations occurred in the South, the census region with the largest population. However, the Northeast had the highest rate per 100,000 population (15.8), 53% higher than in the Midwest (10.3) or West (10.3); the South had an intermediate rate (12.1). (All-cause hospitalization rates are similar in all regions except the West, where rates were about 20% lower). Among endocarditis patients, substance use and HIV were most common in the Northeast and least common in the Midwest.

**Table 2:** Demographic, Other Patient, and Hospital Characteristics of Patients Hospitalized with Endocarditis, 1998–2009.

Characteristic	Percent
Age	
<18	1.9
18–44	20.0
45–59	24.4
60–69	17.4
>69	36.4
Male	57.7
Race/ethnicity*	
White	69.4
Black	17.1
Hispanic	8.4
Asian or Pacific Islander	2.0
Native American	0.6
Other	2.6
Insurance**	
Private	24.2
Medicare	53.3
Medicaid	13.8
Self-pay/no charge	5.9
Other	2.9
Region	
Northeast	24.4
South	36.2
Midwest	19.8
West	19.6
Mean income of zip code of patient’s residence	
Poorest quartile	28.3
Second quartile	25.4
Third quartile	23.4
Wealthiest quartile	23.0

* Information on patients’ race was not reported for 21.3% of patents.

** Expected primary payer as indicated by hospital.

**Figure 1:**
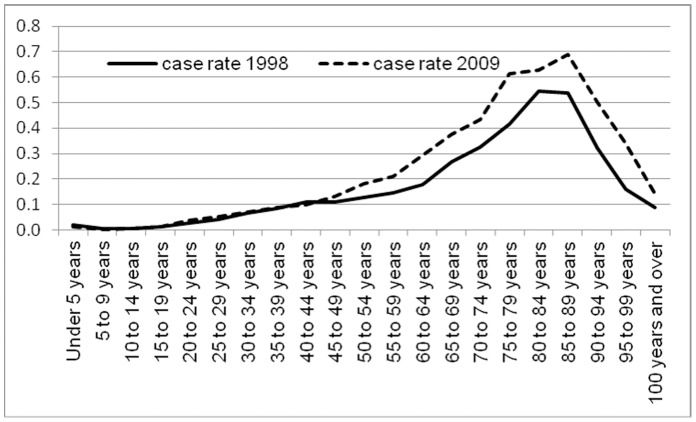
Infective endocarditis hospitalizations: rate per thousand population according to 5 year age groups: 1998 and 2009.

Table 3 displays comorbidities and predisposing factors. Between 1998 and 2009 the mean age of endocarditis patients rose from 58.6 to 60.8 years. This shift reflects stable incidence rates in younger populations, and substantial increases in all age groups older than 50 (Figure1). Cases with comorbid drug use, HIV (or both) fell both absolutely, and as a share of all endocarditis hospitalizations; the proportion with HIV fell from 4.8% to 1.5%, while the share with a diagnosis of drug use (narcotics, cocaine or amphetamines) fell from 9.7% to 4.7%. The proportion of patients with a pre-existing cardiac valve replacement, implant or indwelling device rose 42%, from 13.3% in 1998 to 18.9% in 2009. The proportion on chronic dialysis was 4.4%, peaking in 2006 before declining.

**Table 3:** Comorbidities and Predisposing Factors Among Cases of Diagnosed Infective Endocarditis, 1998–2009.

Year	Mean Age of Patients	HIV (%)	Drug use (%)*	Chronic Dialysis (%)	Cardiac Device, Implant or Graft (Including valve)(%)	Any Pre-existing Cardiac Abnormality** (%)
1998	58.7	4.8	9.7	1.8	13.3	23.7
1999	58.8	4.6	9.6	3.0	13.6	22.8
2000	59.8	3.2	7.1	2.6	14.4	23.8
2001	59.6	3.3	8.4	3.1	14.4	24.7
2002	59.4	3.4	8.7	3.6	14.4	24.6
2003	59.2	2.5	8.2	3.9	15.0	25.4
2004	58.9	2.2	9.0	4.7	14.8	24.9
2005	60.0	2.2	8.8	5.8	15.5	25.6
2006	59.5	2.9	8.4	7.5	15.6	26.4
2007	60.6	2.3	7.6	6.6	16.0	27.0
2008	60.9	1.8	5.4	4.0	17.8	26.3
2009	60.8	1.5	4.7	4.6	18.9	27.7
12 Year Total	59.7	2.8	7.8	4.4	15.5	25.4

* Narcotic, amphetamine, or cocaine use

** Includes a coded congenital anomaly, rheumatic and syphilitic heart disease, or the presence of a cardiac device, implant, or graft.

### Microbiology

An organism was coded for 62.3% of IE discharges in 1998, rising to 69.4% in 2009 (Table 4). Because most case series report rates of culture-negative endocarditis of less than 10%, it seems likely that about a quarter of endocarditis patients in the NIS had known microbiology that was not coded in the discharge summary. Patients with no coded organism were similar to patients with a bacteriologic diagnosis according to age, race, predisposing factors and most complications. However, they were slightly more likely to undergo valve replacement, to live in the South or in a poor zip code, be hospitalized in a rural hospital, and be female. Multiple organisms were mentioned in 4.2% of discharge records, a number that exceeds previous estimates of polymicrobial endocarditis. Some of these probably represent a second infection (e.g. pneumonia) during an endocarditis hospitalization. The data below (and in Table 4) should be interpreted with these caveats in mind.

**Table 4:** Proportion of Cases of Infective Endocarditis Due to Specific Organisms, 1998–2009.

Organism	% of All Cases	% of Cases With Known Organism
Staphylococcus aureus*	28.7%	44.2%
Other staphylococci	6.7%	10.3%
Streptococci	24.7%	38.0%
Group A	0.7%	1.1%
Group B	1.3%	2.0%
Group C	0.1%	0.2%
Group D (enterococci)	6.4%	9.8%
Group G	0.2%	0.3%
S. pneumoniae	0.4%	0.6%
Gram negative rods	5.6%	8.6%
E. coli	1.7%	2.6%
Pseudomonas	1.2%	1.8%
Proteus	0.2%	0.3%
Serratia	0.1%	0.2%
H. influenza	0.2%	0.3%
Klebsiella	0.6%	0.9%
Meningococci	0.01%	0.02%
Gonococci	0.02%	0.03%
Anaerobes	0.4%	0.6%
Q Fever	0.04%	0.1%
Mycoplasma	0.01%	0.02%
S. typhi	<0.01%	<0.01%
Candida	1.0%	1.5%
Other fungi	<0.01%	<0.01%
Psittacosis	<0.01%	<0.01%
Other bacteria (species unknown or unspecified)	2.2%	3.4%
No microbiology coded	35.1%	NA
More than one organism coded**	4.2%	6.5%

* In 2009, 53.3% of staphylococci were coded as “methicillin resistant” (MRSA). MRSA was not specifically coded in prior years.

** Some cases may represent a second, non-endocarditis infection.

The most common organism was Staphylococcus aureus, mentioned in 28.7% of discharge summaries (44.2% of summaries mentioning any organism), a proportion that increased by nearly one third, from 24.4% in 1998 to 32.0% in 2009. It was particularly common among patients with substance use, mentioned in 46.9% of those cases. Methicillin resistant S. aureus (MRSA) accounted for 53.3% of S. aureus among endocarditis discharges in 2009, the only year in which NIS identified methicillin-resistance. Other staphylococci were mentioned in 6.7% of discharges. Other organisms showed no marked trends. Streptococci were mentioned in 24.7% of discharge summaries, of which more than one quarter were labeled Group D (enterococci).

There was no change in the proportion (nor in overall IE incidence) of cases attributable to non-enterococcal streptococci (i.e. cases that might be affected by antibiotic prophylaxis) coincident with the 2007 change in recommendations regarding prophylaxis (p = .53 for comparison between rate trend for non-enterococcal streptococci, 1998–2006 vs. 2008–2009).

Gram-negative bacilli were mentioned in 5.6% of endocarditis patients’ discharge summaries, and Candida species in 1.0%.

### Outcomes

Table 5 summarizes endocarditis outcomes; 14.5% of patients with a diagnosis of IE died in-hospital. Changes in the death rate over the 12 years of the study were negligible. The rate of valve replacement during the hospital stay did not change (p = .92), averaging 9.6% of patients; 4.6% of mitral valves, and 6.1% of aortic valves were replaced (some patients underwent both procedures).

**Table 5:** Outcomes for Patients Hospitalized with Infective Endocarditis, 1998–2009.

Year	Death (%)*	Stroke or CNSInfection (%)	Cardiac ValveReplacement (%)	MyocardialInfarction (%)	Died, stroke, CNS infection or MI (%)
**1998**	13.30	9.18	8.84	3.17	20.28
**1999**	15.29	9.04	9.67	3.68	22.25
**2000**	15.17	9.46	9.68	4.59	23.26
**2001**	16.16	9.43	11.06	4.79	23.79
**2002**	15.86	10.19	9.61	5.63	24.74
**2003**	15.77	10.33	10.77	5.97	25.48
**2004**	14.61	10.50	9.36	5.57	24.41
**2005**	13.89	11.08	8.84	5.72	24.08
**2006**	14.11	10.71	9.02	5.98	24.16
**2007**	12.90	12.12	8.79	5.79	24.09
**2008**	14.61	12.44	9.90	6.82	26.22
**2009**	13.00	13.25	9.91	6.78	25.60
**12 Year Total**	**14.49**	**10.81**	**9.61**	**5.51**	**24.19**

* Patients transferred to a second acute care hospital were excluded from both the numerator and denominator in calculating the death rate, but not for other or composite endpoints.

Serious neurological complications (stroke or CNS infection), MI, and the composite endpoint of these plus death, all increased. The composite endpoint was met by 25.6% of discharged patients in 2009, up from 20.3% in 1998. MI rates more than doubled from 3.2% to 6.8%, while serious neurological complications rose from 9.2% to 13.3%. Overall, a stroke occurred during 9.0% of endocarditis hospitalizations, CNS infection during 2.1%, convulsions during 3.8%, and encephalopathy during 2.2% of the hospital stays. ARF was diagnosed in 18.0% of patients.

Patients with S. aureus had significantly higher mortality (17.4%) than those with other (or no) specified causative organisms (11.3%), and higher rates of CNS infection (3.9% vs. 1.4%) and ARF (22.7% vs. 16.0%). The difference in stroke rates was small (9.6% vs. 8.8%, p = .0005).

Other predictors of mortality included: any neurological complication (23.3%), especially stroke (28.5%); ARF (27.1%); and MI (31.6%). Patients over 70 had the highest mortality (16.1%), while those 18–44 had the lowest (4.8%); 8.5% of children died. Mortality was not notably affected by HIV, pre-existing valvular abnormalities, or valve replacement during hospitalization, but was lower among patients with substance use (8.3%).

## Discussion

The incidence of hospitalization for endocarditis in the U.S. is 12.7 per 100,000 annually. Even accounting for the fact that multiple hospitalizations might occur during a single episode of IE, the incidence of this illness appears substantially higher than previously reported [5], [8]. The age adjusted 2.4% annual increase in hospitalizations between 1998 and 2009 is in keeping with reports of rising rates of sepsis and septicemia during this period [12]. However, it represents an acceleration of the trend in IE incidence observed in a review of twelve small population-based studies in Europe and Olmsted County, Minnesota [8]. That review found rates (per 100,000) of 1.4–1.8 during the 1970s and 1980s (with one outlier), rising to 2.2–4.9 during the 1990s.

The IE patients we identified resemble those in a 2000–2005 prospective cohort study of 2,781 patients in 25 countries (ICE-PC) where 25% of all cases (and 38% of US patients) had health care-associated infections [5], [13]. Several epidemiologic features of our patients – their advanced age, the male predominance, the prevalence of co-morbid illness and cardiac implants, grafts and devices, and the dominant role of S. aureus (and, in 2009, MRSA) – suggest that health care-associated infections were frequent.

Remarkably, co-morbid drug use and HIV, although probably under-reported, fell between 1998 and 2009. Perhaps harm reduction programs stressing needle hygiene, and a shift from intravenous to oral opioid use underlie this decrease [14].

The rising age of endocarditis patients explained few of our findings. Although death and MI rates were higher among the elderly, time trends for these outcomes did not vary by age. The proportion of IE hospitalizations attributed to S. aureus was slightly higher among the non-elderly (in keeping with findings from the ICE-PC cohort [15]) and rose faster. The rise in neurologic complications occurred largely among those under 70, and this group accounted for the entire decline in HIV and substance use-associated cases.

We found marked geographic variation in IE hospitalization rates, with higher rates in poorer communities and in the Northeast. This finding remains unexplained; differences in all-cause hospitalization rates and mentions of substance use and HIV were small. The ICE-PC study also documented marked geographic variation (by continent) in the epidemiology of IE [5].

The geographic variability we observed may, in part, explain the lower estimates of IE incidence in earlier studies. For instance, a widely-cited U.S. study assessed IE incidence in Olmstead County, Minnesota, a region in the Midwest with a relatively low poverty rate that may not be nationally representative.

Despite changes in recommendations that greatly reduced the use of antibiotic prophylaxis for dental and other procedures around 2007 [16], [17], we found no coincident inflection in the time trend of total IE hospitalizations or of the subset caused by non-enterococcal streptococci. These reassuring findings mirror those from England and Wales [18].

Endocarditis outcomes didn’t improve during the 12-year period we studied - perhaps due to the growing predominance of S. aureus infections. Severe complications increased. At 14.5%, mortality was lower than the 18% rate in the ICE-PC study, which oversampled referral hospitals [5]. Our analysis probably undercounts deaths; LOS was only 15 days, and some patients undoubtedly died while completing therapy in convalescent settings. In one study, 12% of IE patients died within one year and a third by four years after discharge [19].

The rate of valve replacement surgery we observed is far lower than the 48.2% who underwent surgical therapy in the ICE-PC cohort, perhaps because a larger proportion of the ICE-PC patients were cared for in referral centers [5].

Recent studies report similar neurologic complication rates to those we found [5], [20]–[22]. In our sample, 9% of IE patients suffered strokes versus 7.8% to 21% in others’ series. We found low rates of meningitis and brain abscess, only 2.1%; prior studies reported meningitis in 5% to 16.5% of patients and brain abscess in 0% to 4% [20], [23]. Our finding that CNS infections and stroke were more common with S. aureus infection confirms prior findings [20], [21], [23]. Some [21], but not all [20] previous studies have, like ours, found an association between neurologic complications and mortality.

Inflation-adjusted costs of care doubled between 1998 and 2009, rising at the same rate as costs for other diagnoses despite the absence of important therapeutic innovations for IE. The cost increase cannot be attributed to IE patients’ increasing age; costs were lowest for the oldest age group.

Several caveats apply to our analysis. While the NIS data offers advantages of sample size, wide geographic coverage, the ability to assess time trends, and lack of referral bias, ascertainment of diagnoses depends upon both diagnostic and coding rigor. The discharge summaries from which the NIS data are compiled serve, in part, as the basis for hospital payment, and are hence subject to regulation and audit. Hospitals are supposed to code all diagnoses treated during the stay. Major diagnoses and procedures (e.g. endocarditis or valve replacement) are unlikely to go uncoded, or to be affected by changes in coding practices during our study period. Conversely, regulations proscribe coding for inactive problems and those not treated during the hospital stay (e.g. a history of IE); hospitals that billed for IE in the absence of active infection would be subject to sanctions.

To explore whether over-diagnosis might explain the high incidence of endocarditis we found, we examined hospitalizations in which the IE diagnosis was not listed first. In such cases the most common first diagnoses were conditions closely associated with IE such as sepsis or “complication of an implanted device”. Similarities in patient demographics, organisms, outcomes and cost between patients whose IE was coded as a first vs. subsequent diagnosis suggests that our case ascertainment strategy accurately reflects physicians’ diagnoses of IE.

However, predisposing factors or complications are probably under-coded, since clinicians and coders might not consider them important to patients’ care or to reimbursement. Hence, the NIS likely under-reports conditions such as intermittent drug use, anatomically abnormal but functionally adequate heart valves, the presence of a pacemaker, or the occurrence of a single seizure or brief bout of ARF.

The NIS does not specify the sequence of events during a hospitalization. Hence, we cannot ascertain whether a valve surgery that occurred during an IE hospitalization represented a cause or a treatment of the infection. However, few cases of early prosthetic valve IE acquired during surgery would come to light prior to discharge.

Our hospital-based analysis may underestimate population IE incidence. Some IE patients may die prior to a hospital admission or (rarely) be diagnosed and treated without being admitted. Similarly, we could not identify hospitalized cases whose IE was never identified and who either died or were cured by treatment for another condition such as osteomyelitis. Our downward adjustment to account for transfers to another hospital probably causes underestimation of the number of unique cases; it assumes that all cases were coded as having IE at the receiving hospital.

On the other hand, as emphasized above, some patients are probably double-counted in our analysis because they underwent more than one hospital admission in the course of a single episode of IE. We would expect such readmissions to be more frequent at hospitals which often complete treatment in the outpatient setting. However, recent IE case series from such institutions indicate that such readmission are uncommon −3% to 5% of all admitted IE patients [24], [25]. In any event, it seems unlikely that readmissions of outpatient-treated cases accounts for the sharp upswing in IE admissions we observed. While we’re unaware of any national data on trends in the frequency of outpatient IE treatment, the stable length of IE inpatient stay during the course of our study argues against a large increase in outpatient therapy.

The major caveat is that, unlike case series that include only verified and adjudicated diagnoses, we examined clinically diagnosed IE. Hence, our findings reflect the over-diagnosis (and perhaps under-diagnosis) of IE in actual clinical practice, where physicians must sometimes presumptively treat IE despite inconclusive blood culture and echocardiographic data. In von Reyn’s sentinel study [25], the first to apply the now standard [26], strict definitions of endocarditis, and in the recent prospective ICE-PS study [5], only 85% of clinically diagnosed cases met strict criteria for IE. A 15% downward adjustment would reduce our incidence estimate to 10.8 IE hospitalizations annually per 100,000, a figure that is still about twice previous U.S. estimates. Unless diagnosis accuracy is deteriorating, over-diagnosis could not explain the temporal increase we observed.

IE is much more common than previously believed and continues to increase due to the increasing incidence among individuals 50 and older. Rising numbers of cases of IE are due to S. aureus or associated with cardiac devices and implants, and a falling number is linked to drug use. These trends point to the growing importance of patient safety measures to minimize health care-acquired infection. The marked geographic variation we observed remains unexplained, suggesting the salience of future research on Staphylococcal virulence factors as well as environmental and social factors associated with IE.

### Appendix

#### Identifying causative organisms

Although the ICD-9-CM includes diagnostic codes identifying specific causative organisms for infectious diseases, only about two-thirds of records carrying a diagnosis of endocarditis included an indication of a causative organism. We considered a patient with endocarditis to have a staphylococcus aureus endocarditis if any of the following diagnostic codes were included among his/her diagnoses: 03811, 03812, 04111, 04112. Other staphylococci were identified using the following diagnostic codes: 0381, 03810, 03819, 0411, 04110, 04119. Streptococcal endocarditis was identified by the presence of diagnostic codes 0380, 0382, 0410, 04100, 04101, 04102, 04103, 04104, 04105, 04109; enterococcal endocarditis by 04104; gram negative endocarditis by 04185, 0384, 03840, 03841, 03842, 03843, 03844, 03849, 0413, 0414, 0415, 0416, 0417; anaerobic endocarditis by 0383, 04182, 04183, 04184; other bacterial endocarditides by 0388, 04189, 04181, 09884, 0830, 0020, 03642; and fungal endocarditis by 1160, 1154, 11281.

#### Identifying comorbidities, predisposing factors and complications of endocarditis

We identified comorbidities and predisposing factors to endocarditis using the following ICD-9-CM diagnostic codes: For HIV infection: 042, v08, 07953; for opiate, cocaine or amphetamine abuse: 3055, 3056, 3057, 30550, 30560, 30570, 30551, 30561, 30571, 30552, 30562, 30572, 3040, 30400, 30401, 30402, 3042, 30420, 30421, 30422, 3044, 30440, 30441, 30442, 3047, 30470, 30471, 30472, 3048, 30480, 30481, 30482, 3049, 30490, 30491, or 30492; and for patient who were on renal dialysis prior to their endocarditis hospitalization V451 and V4511. We identified pre-existing cardiac abnormalities that predispose to infective endocarditis using the following diagnostic codes: for congenital cardiac anomalies: 7464, 7461, 7466, 74600, 74601, 74602, 74609, 7462, 7463, 7464, 7465, 7465, 7467, 7469, 7468, 74681, 74682, 74683, 74684; for chronic rheumatic heart disease: 3950, 3951, 3952, 3953, 3959, 394, 3940, 3941, 3942, 3949, 396, 3960, 3961, 3962, 3963, 3968, 3969, 397, 3970, 3971, 3979, 398, 3989, 39890, 39891, 3989, 39899; for syphilitic heart disease: 0932, 09320, 09321, 09322, 09323, 09324; and for the presence of a cardiac device, implant, or graft: 9960, 99600, 99601, 99602, 99604, 99661, v433, v4501, v450, v4500, v4502.

We used the following procedure codes to identify patients who underwent cardiac valve replacement during their endocarditis hospitalization: 3521, 3522, 3523, 3524, 3520, 3525, 3526, 3527.

We classified patients as having a possible complication of endocarditis using the following diagnostic codes: for acute renal failure 5845, 5847, 5848, 5849, 5846, 584; for myocardial infarction 41000, 41001, 41002, 41010, 41011, 41012, 41020, 41021, 41022, 41030, 41031, 41032, 41040, 41041, 41042, 41050, 41051, 41052, 41061, 41062, 41070, 41071, 41072, 41080, 41081, 41082, 41090, 41091, 41092; for stroke 430, 431, 4320, 4329, 43301, 43311, 43321, 43331, 43381, 43391, 43401, 43411, 43491; for central nervous system abscess or meningitis 320, 3200, 3201, 3203, 32081, 32082, 32089, 3208, 3209, 11283, 1142, 11501, 11511, 11591, 3240, 3241, 3249; for encephalopathy 3483, 34831, 34830, 34839; and for convulsions 7803,78039 (excludes epilepsy).
